# The potential value of plasma receptor interacting protein 3 in neonates with culture-positive late-onset sepsis

**DOI:** 10.1186/s12879-021-06636-0

**Published:** 2021-09-06

**Authors:** Chuchu Gao, Zongtai Feng, Lixia Wang, Xingxing Zhao, Kai Fu, Shurong Ma, Zuming Yang, Sannan Wang, Shenglin Yu

**Affiliations:** 1 Department of Neonatology, Children’s Hospital of Soochow University, Suzhou, 215025 China; 2 Department of Neonatology, The Affiliated Suzhou Hospital of Nanjing Medical University (Suzhou Municipal Hospital), Suzhou, 215002 China; 3 Department of Urology, The Second Affiliated Hospital of Soochow University, Suzhou, 215006 China

**Keywords:** Late-onset sepsis, Neonate, Receptor interacting protein 3

## Abstract

**Background:**

Late-onset sepsis (LOS) is a systemic inflammatory response syndrome in neonates, and the molecular mechanism of LOS is incompletely characterized. The purpose of this study was to explore the potential value of receptor interacting protein 3 (RIP3) in LOS.

**Methods:**

63 neonates with LOS supported by positive culture and 79 neonates without sepsis were enrolled in this study from September 2019 to March 2021. Plasma RIP3 was detected by enzyme-linked immunosorbent assay (ELISA) and assessed along with the whole blood hypersensitive C-reactive protein (hs-CRP) level and platelet count (PLT). Differences in RIP3, hs-CRP and PLT between the two groups were compared. Changes in the three indicators in sepsis were also observed after treatment. The diagnostic value of indicators for LOS was evaluated by receiver operating characteristic (ROC) curve analysis.

**Results:**

In the sepsis group, RIP3 and hs-CRP levels were significantly higher than those in the control group (RIP3, *p* < 0.0001; hs-CRP, *p* < 0.0001), and PLT was significantly lower than that in the control group (*p* < 0.0001). After treatment, RIP3 and hs-CRP levels among septic survivors were significantly decreased (*p* < 0.0001) and PLT significantly improved (*p* = 0.0216). With RIP3 > 15,845.19 pg/mL, hs-CRP > 5.00 mg/L, and PLT < 204.00 × 10^9^/L as the positive criteria, the sensitivity values of the three indicators in the diagnosis of LOS were 69.8%, 60.3%, 60.3%, respectively, and the specificity values were 92.4%, 96.2%, 79.8%, respectively. The combination of RIP3, hs-CRP and PLT had a sensitivity of 77.8% and specificity of 97.5%.

**Conclusions:**

RIP3 may contribute to the early diagnosis of LOS and monitoring of treatment effect. The combined detection of RIP3, hs-CRP and PLT may be more effective than individual detection in the diagnosis of LOS.

**Supplementary Information:**

The online version contains supplementary material available at 10.1186/s12879-021-06636-0.

## Background

Late-onset sepsis (LOS), defined as neonatal sepsis occurring after 72 h of age, is a major cause of morbidity and mortality in neonatal populations [1–3]. The early diagnosis of LOS is challenging because its clinical features are highly variable and may be confused with non-infectious disorders [4, 5]. Missed identification and delayed treatment can rapidly lead to multiple organ dysfunctions [6–8]. Moreover, some survivors of LOS may have behavioral and neurocognitive dysfunction, mood disorders, and a low quality of life, which place a large burden on the medical system and society [2, 9]. Prompt diagnosis of LOS is crucial for improving outcomes and prognoses. However, the early diagnosis of LOS remains a serious global problem despite major efforts.

Isolation of causative microorganisms by blood culture is still the gold-standard for the diagnosis of LOS [10], but it has many limits in practical application due to its long time consumption and low sensitivity. In addition, some traditional nonspecific indicators, such as white blood cell count (WBC), platelet count (PLT), C-reactive protein (CRP) and procalcitonin (PCT) do not have sufficient sensitivity or specificity for early identification. Over the past few years, distinct biomarkers, including cell surface receptors, bacterial surface antigens and genetic biomarkers, have been evaluated. Protein biomarkers, cytokines and chemokines are receiving a lot of attention for the identification of neonatal sepsis [11, 12]. However, to date, there is no single ideal biomarker that has high sensitivity or specificity [1, 11].

Receptor interacting protein (RIP) 3 belongs to the RIP kinase family [13]. Recent evidence suggests that RIP3-dependent necroptosis is activated in a variety of diseases [14–16]. Stimulated by biological or physicochemical factors, the tumour necrosis factor receptor binds to ligands and recruits Fas-associated protein with death domain (FADD), RIP1 and caspase-8 to form complex I, which promotes apoptosis [16]. When caspase-8 activity is inhibited, RIP1 binds to RIP3 and recruits FADD and caspase-8 to form complex II, which suppresses apoptosis and turns into the programmed necrosis pathway, leading to cell swelling and rupture [17]. A large number of damage-associated molecular patterns (DAMPs) are released, triggering a strong inflammatory response. To date, studies on the predictive value of RIP3 in sepsis involve mainly adult patients [18, 19]. However, the role of RIP3 has not been comprehensively reported in neonatal sepsis.

In this pilot study, we measured the plasma levels of RIP3 in children with LOS and compared them with hypersensitive C-reactive protein (hs-CRP) and PLT to explore its potential value for LOS diagnosis.

## Methods

### Patients and study design

This study recruited patients from September 2019 to March 2021 in the Department of Neonatology of Suzhou Municipal Hospital. Infants who were diagnosed with LOS were included. The diagnosis was based on the criteria published by the National Institute of Child Health and Human Development (NICHD) [7]. In brief, the definition of LOS includes sepsis onset after 72 h of life, with positive blood culture and either completion of ≥ 5 days of antibiotics or death before the course is completed. Other inclusion criteria were as follows: (a) age < 28 days and (b) routine blood examinations, with hs-CRP measurements and blood cultures performed before the use of antibiotics. Neonates admitted for non-infectious diseases, including premature infants whose mothers had severe eclampsia or mental disorders, hypoglycaemia or breastfeeding-related jaundice, were included as controls. The baseline maternal and perinatal period data in the control group were well balanced with those in the sepsis group. The exclusion criteria included early-onset sepsis (EOS) and suspected sepsis but with negative culture results. Additionally, infants who had incomplete information or discontinued therapy due to their guardians signing a request to give up treatment were also excluded.

This study was approved by the Medical Ethics Committee of Suzhou Municipal Hospital. All cases were conducted with informed consent by the legal guardians.

### Sample collection and data recording

General information, including sex, gestational age, delivery mode, APGAR score, amniotic fluid, birth weight, pregnancy status and age at blood collection, was collected.

Before antibiotic treatment, 3 mL of peripheral blood was collected from septic infants. 2 mL of each sample was stored in culture bottles (Becton, Dickinson and Company [BD] BACTEC Peds Plus/F and BD BACTEC Lytic/10 Anaerobic/F) and sent to the microbiology laboratory for blood culture using a BACTEC fx200 blood culture system (BD, Franklin Lakes, USA). The other 1 mL in each sample was gathered in an ethylenediaminetetraacetic acid (EDTA)-containing tube, tested in the clinical laboratory for hs-CRP and routine blood analysis by immune turbidimetry and with an XE-2100 blood cell analyser (Sysmex Corporation, Kobe, Japan), respectively, and then centrifuged at 1 610×*g* for 10 min. The supernatant was collected and stored at -80℃ for RIP3 detection. After treatment (approximately 7 days of effective antibiotic treatment), 1 mL of the blood sample was collected for the hs-CRP measurement and routine blood tests. In the control group, 1 mL of the blood sample was obtained to detect hs-CRP and perform routine blood tests. Plasma was separated and stored by the same method.

The level of RIP3 in plasma was detected using enzyme-linked immunosorbent assay (ELISA) kits (CUSABIO Technology LLC, Wuhan, China) following the manufacturer’s instructions.

### Statistical analysis

The normality of distribution of quantitative data was determined with the Kolmogorov–Smirnov test. Data with a normal distribution are described as the mean ± standard deviation and were compared between groups using a t-test. Quantitative data with a non-normal distribution are expressed as the median and interquartile range and were compared between groups using the nonparametric Mann–Whitney *U* test. Categorical data are expressed as numbers (percentages) and were compared between groups using the chi-square test or Fisher’s exact test. Data collected from the research were analysed by SPSS version 22.0 (SPSS, Chicago, IL, USA). GraphPad Prism version 8.3.0 (GraphPad, San Diego, CA, USA) was used to prepare graphs. A receiver operating characteristic (ROC) curve was drawn by MedCalc version 18.2.1 (MedCalc, Ostend, Belgium) to determine the best cut-off value of plasma RIP3 and assess the efficacy of a single index and combined indexes for the prediction of neonatal sepsis. A difference with a *p* value of less than 0.05 was considered statistically significant.

## Results

### Patient characteristics

In the initial results, we excluded eight infants with EOS, twenty-two neonates with suspected sepsis but with negative culture results, eight infants who had incomplete information and three infants whose guardians signed a request to give up treatment within seven days of onset. Eventually 142 infants were included in this study. Among them, 63 neonates with sepsis supported by positive culture were included in the sepsis group, with a median gestational age of 32.1 (30.1–36.6) weeks. 79 neonates without sepsis were included in the control group, with a median gestational age of 33.3 (30.4–35.3) weeks. In the sepsis group, one child died the next day. There were no statistically significant differences in maternal factors, demographic characteristics, clinical properties or age at blood collection between the two groups (Additional file 1: Table S1).

### Comparisons of RIP3, hs-CRP and PLT between the sepsis group and the control group

**Fig. 1:**
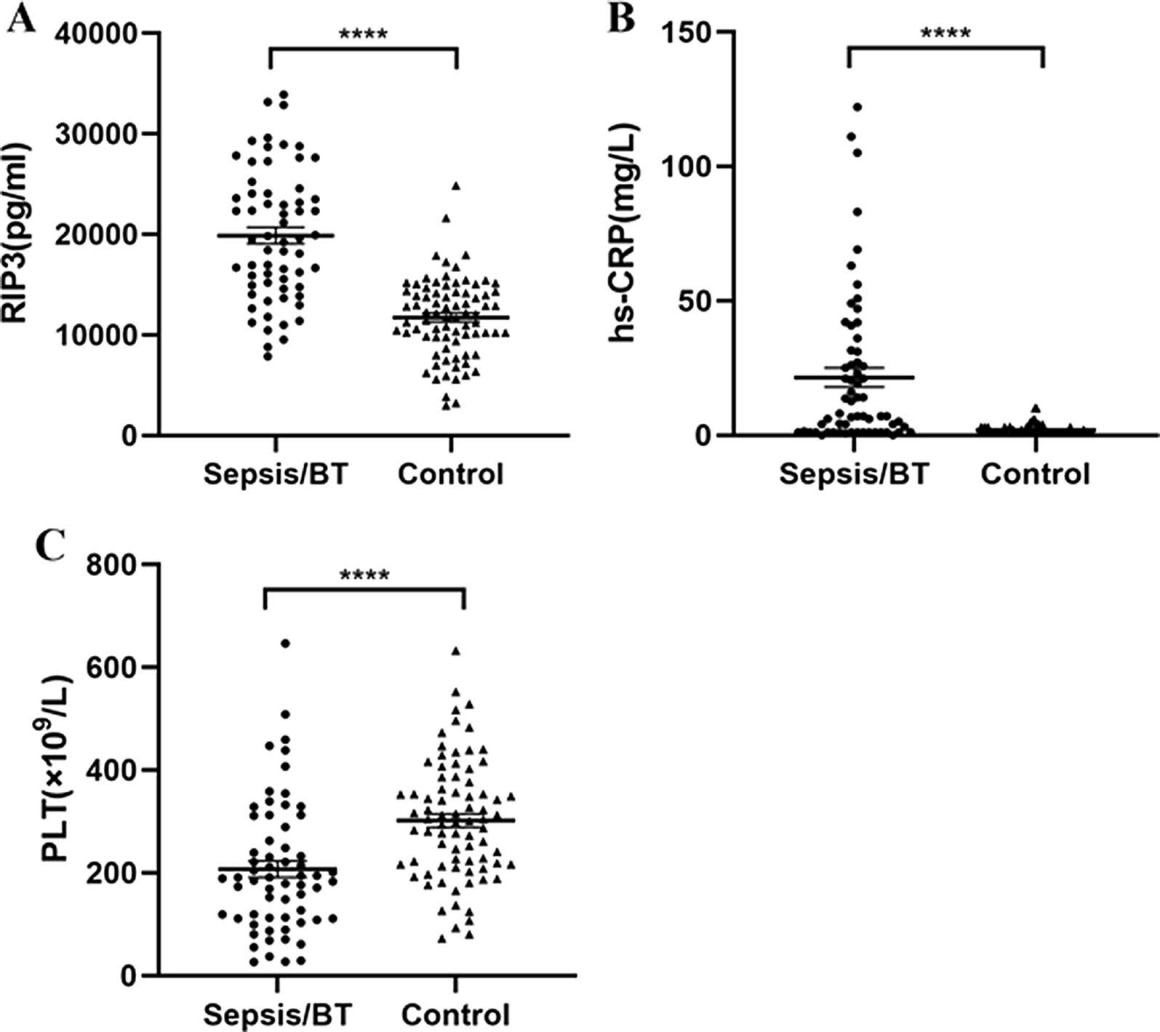
Quantification of RIP3, hs-CRP and PLT levels in the sepsis group and control group. *BT* before treatment. **** *p* < 0.0001

The RIP3 and hs-CRP levels and PLT in the sepsis group before treatment were 19,861 ± 6542 pg/mL, 7 (1–31) mg/L and 189 (111–289) × 10^9^/L, respectively, and those in the control group were 11,729 ± 3959 pg/mL, 1 (1–2.1) mg/L and 301 (216–376) × 10^9^/L, respectively. Further comparative analysis showed that RIP3 and hs-CRP levels in the sepsis group were significantly higher than those in the control group (*p* < 0.0001, Fig. 1a and p < 0.0001, Fig. 1b), and that the levels of PLT were significantly lower in the sepsis group than those in the control group (*p* < 0.0001, Fig. 1c).

### Comparisons of RIP3, hs-CRP and PLT in the sepsis group before and after treatment

**Fig. 2:**
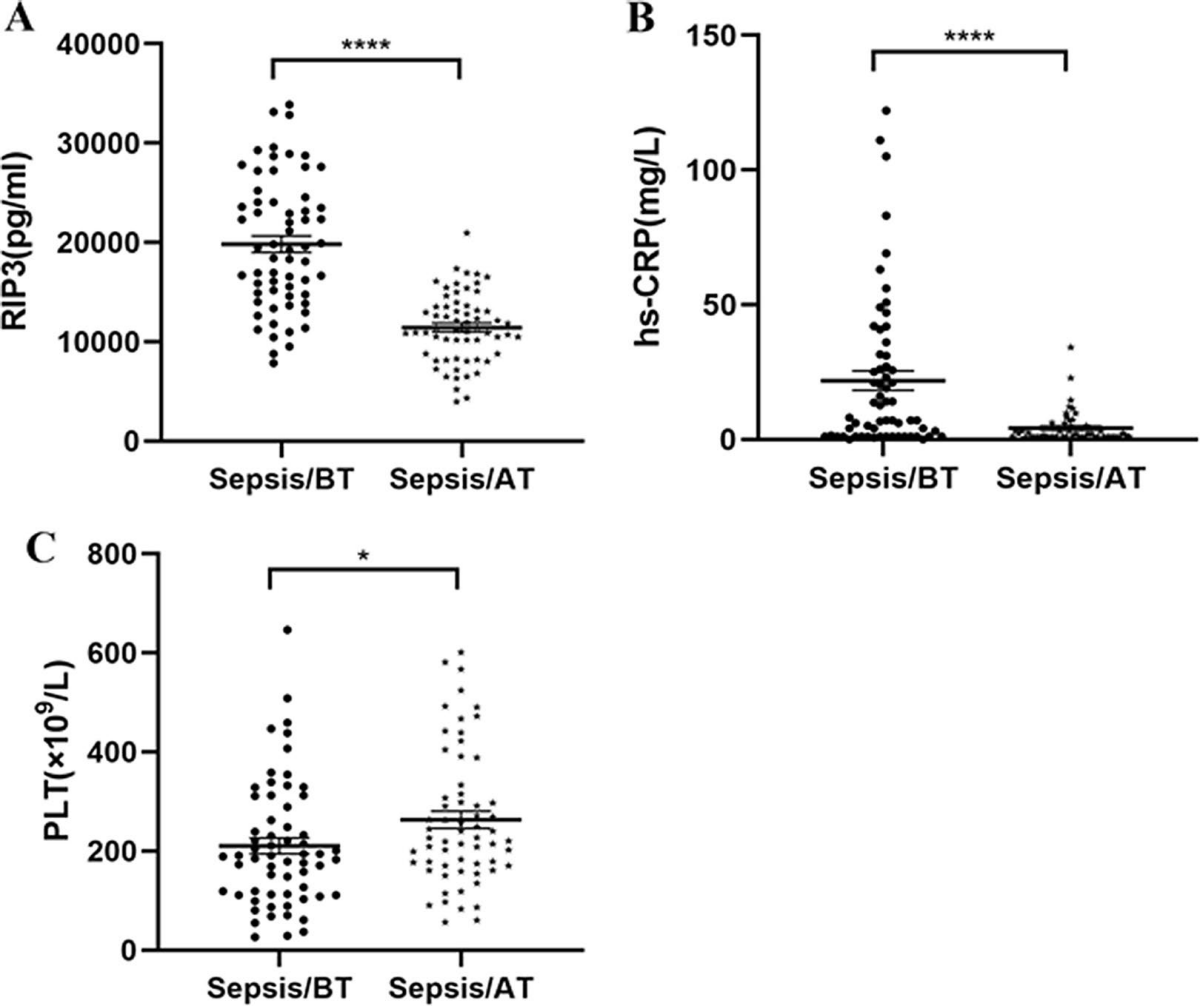
Quantification of RIP3, hs-CRP and PLT levels in the sepsis group and control group. *BT* before treatment, *AT* after treatment. **** *p* < 0.0001, * *p* < 0.05

In the sepsis group, the RIP3 and hs-CRP levels and PLT before treatment were 19,821 ± 6588 pg/mL, 7.5 (1–31.1) mg/L and 190 (111.8–294.5) × 10^9^/L, and those after treatment were 11,432 ± 3493 pg/mL, 2.2 (1–4.3) mg/L and 226.5 (170–319.5) × 10^9^/L. Furthermore, comparative analysis showed that after effective treatment, RIP3 and hs-CRP levels significantly decreased compared with those before treatment (*p* < 0.0001, Fig. 2a and p < 0.0001, Fig. 2b). PLT was significantly increased compared with that before treatment (*p* = 0.0216, Fig. 2c).

### ROC curve analysis of the predictive value of RIP3, hs-CRP and PLT for neonatal sepsis

**Fig. 3:**
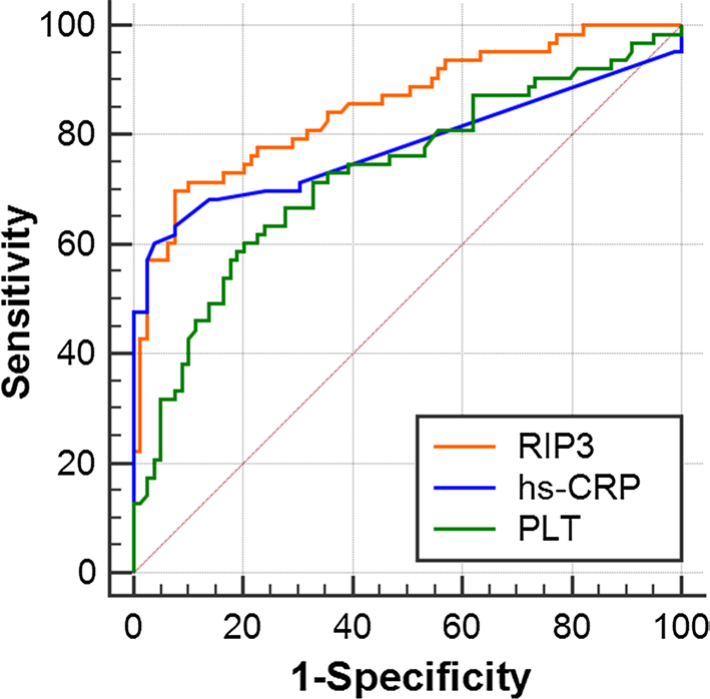
ROC curve for the ability of RIP3, hs-CRP and PLT quantification to predict neonatal sepsis

ROC curves for the ability of RIP3, hs-CRP and PLT to predict neonatal sepsis were constructed (Fig. 3). The optimal cut-off value for the plasma RIP3 level was 15,845.19 pg/mL with an area under the curve (AUC) of 0.854. The sensitivity of RIP3 was 69.8%, and the specificity was 92.4%. Further comparative analysis showed that the AUC, sensitivity, negative predictive values and Youden index of hs-CRP and PLT were smaller than those of RIP3 (Table 1).

**Table 1:** Predictive value of RIP3, hs-CRP and PLT for neonatal sepsis

Variable	RIP3(pg/mL)	hs-CRP(mg/L)	PLT(× 10^9^/L)
Cut-off value	15,845.19	5.00	204.00
AUC	0.854	0.780	0.728
Sensitivity	69.8	60.3	60.3
Specificity	92.4	96.2	79.8
PPV	88.0	92.7	70.4
NPV	79.3	75.2	71.6
PPR	9.20	15.88	2.98
NPR	0.33	0.41	0.50
95%CI	0.785–0.908	0.703–0.845	0.643–0.814
YI	0.623	0.565	0.401

*AUC* area under the curve, *CI* confidence interval, *PPV* positive predictive value, *NPV* negative predictive value, *PPR* positive probability ratio, *NPR* negative probability ratio, *YI* Youden index

**Table 2:** Predictive value of biomarker combinations for neonatal sepsis

Variable	RIP3 + hs-CRP	RIP3 + PLT	hs-CRP + PLT	RIP3 + hs-CRP + PLT
AUC	0.892	0.859	0.842	0.897
Sensitivity	77.8	69.8	65.1	77.8
Specificity	97.5	94.9	94.9	97.5
PPV	96.1	91.7	91.1	96.1
NPV	84.6	79.8	77.3	84.6
PPR	30.72	13.79	12.85	30.72
NPR	0.23	0.32	0.37	0.23
95%CI	0.829–0.938	0.790–0.911	0.772–0.898	0.835–0.942
YI	0.753	0.648	0.600	0.753

*AUC* area under the curve, *CI* confidence interval, *PPV* positive predictive value, *NPV* negative predictive value, *PPR* positive probability ratio, *NPR* negative probability ratio, *YI* Youden index

### ROC curves for the use of biomarker combinations to predict neonatal sepsis

**Figure. 4:**
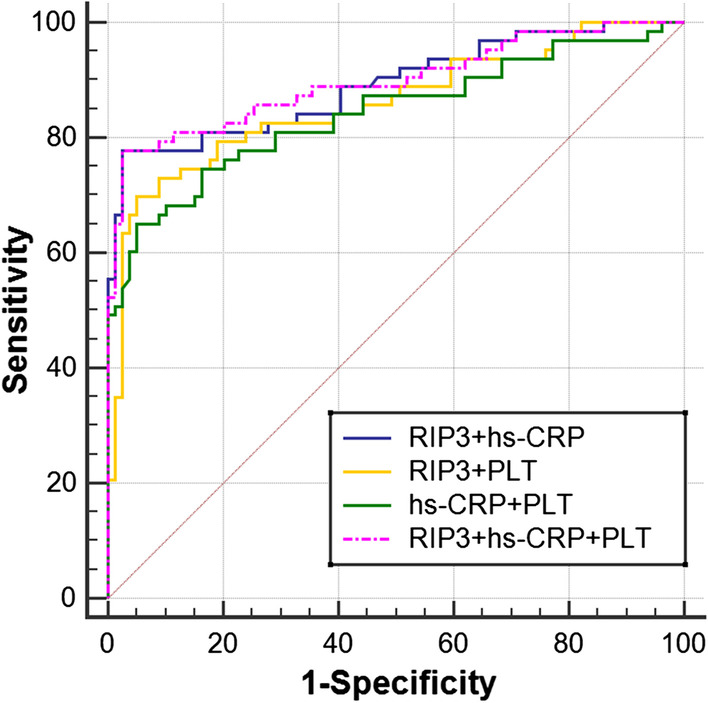
ROC curve for the ability of different combinations of RIP3, hs-CRP and PLT to predict neonatal sepsis

ROC curves were plotted to examine the performance of the combinations of RIP3 + hs-CRP, RIP3 + PLT, hs-CRP + PLT and RIP3 + hs-CRP + PLT (Fig. 4). The AUC, sensitivity, specificity, positive predictive value and negative predictive value of RIP3 + hs-CRP + PLT were 0.897, 77.8%, 97.5%, 96.1% and 84.6%, respectively. These values were higher than those for a single index and other combined indexes (Table 2).

## Discussion

In the pathological state, homeostasis is maintained by apoptosis, which is generally considered to be a noninflammatory biological process [20]. Once this process is inhibited, necrosis can be activated as an alternative pathway to cell death and participates in a series of inflammatory reactions [21]. At present, the mechanisms of necrosis include mainly programmed necrosis, ferroptosis, inflammatory necrosis and other pathways [14, 22–24]. Previous studies have shown the critical role of programmed necrosis in the regulation of systemic inflammatory response syndrome (SIRS). Linde Duprez et al. showed that the deletion of RIP3 reduced the amounts of circulating DAMPs in mice and conferred complete protection against lethal SIRS [25]. Apostolos Polykratis et al*.* had similar results in mice pre-treated with the RIP1 inhibitor necrostatin-1 [26]. Because SIRS is the essence of neonatal sepsis, we speculate that programmed necrosis has specific roles in the pathogenesis of neonatal sepsis.

The latest studies showed that RIP3 protein deficiency attenuated the inflammatory response and organ damage in new-born mice with sepsis [27], suggesting that RIP3 may participate in the pathogenesis of neonatal sepsis. To date, the role of RIP3 in neonatal sepsis has not been reported in the clinic. The current study evaluated the clinical value of plasma RIP3 for LOS. We demonstrated that the levels of RIP3 in the sepsis group before treatment were significantly higher than those in the control group, revealing that plasma RIP3 may be an important biomarker of LOS. Our finding is consistent with reports about the expression of plasma RIP3 in adult patients in the intensive care unit. RIP3 was detectable in the plasma of adult patients with severe sepsis, and the expression of RIP3 in the plasma of patients with sepsis-related death was significantly higher than that in the plasma of survivors, suggesting that the abnormal production or clearance of RIP3 may predict the poor prognosis of sepsis [18]. In a prospective study of 953 patients in five intensive care units, elevated levels of plasma were associated with in-hospital mortality and organ failure [19], which indirectly confirmed that high expression of RIP3 could indicate the adverse consequences of critical illness. We further observed that plasma RIP3 levels in the sepsis group significantly decreased after effective treatment. It is speculated that RIP3 would be helpful to judge the clinical efficacy and prognosis of LOS.

At present, the early diagnosis of neonatal sepsis in practice depends mainly on nonspecific infection indicators such as WBC, PLT, CRP and PCT [28–30]. In this study, hs-CRP and PLT were also investigated and compared with RIP3 to evaluate the value of different indicators in the early diagnosis of LOS. The results showed that the sensitivity, negative predictive values and Youden index of hs-CRP and PLT were lower than those of RIP3, indicating that RIP3 was a biomarker with high diagnostic efficiency. Previous studies have shown that the diagnostic value can be improved by combining different biomarkers [31–33]. According to this point, further comparison of the diagnostic value of multiple combined indicators for LOS was conducted, showing that the sensitivity, specificity, positive predictive value and negative predictive value of RIP3 + hs-CRP + PLT in the diagnosis of LOS were higher than those of RIP3 + PLT and hs-CRP + PLT. Additionally, the AUC of RIP3 + hs-CRP + PLT in the diagnosis of LOS was the highest. It is suggested that the combination of RIP3, hs-CRP and PLT has more robust diagnostic performance for LOS. Furthermore, it is reported that the scoring systems based on physical examination coupled with laboratory findings feature great value in neonatal sepsis predicting [34–36]. Therefore, encompassing physical examination, laboratroy findings plus RIP3 level might be more helpful for LOS early diagnosis, that's why we are intending to focus on this issue in future.

The limitations of this preliminary study are as follows: First, new-borns are prone to iatrogenic anaemia, so the volume of blood collected was relatively small, making the quantity insufficient for detecting multiple indicators. The differences in RIP3 and many other biomarkers, such as PCT and IL, in the diagnosis of neonatal sepsis are unclear. Second, because all new-borns were actively treated with antibiotics immediately after receiving a sepsis diagnosis, there was no patient with untreated sepsis to form a control group. Thus, the clinical intervention factor of antibiotic treatment was not thoroughly studied. Third, there was a lack of monitoring at multiple time points to explore the dynamic expression of different indicators in neonatal sepsis. In addition, due to ethical limitations, it was difficult to obtain blood samples of new-borns under physiological conditions in practice, and the sample size was small; therefore, a comparison of RIP3 expression between different gestational ages and different body weights under physiological conditions was not performed. Last, this is a single-centre preliminary study with a small sample size. Based on the insufficient number of infants with EOS and suspected sepsis but with negative culture results, these cases were excluded from the final statistical analysis, and our findings need to be confirmed by long-term and large-scale multicentre studies.

## Conclusion

RIP3 may contribute to the early diagnosis of LOS and monitoring of treatment effect. The combined detection of RIP3, hs-CRP and PLT may be more effective than individual detection in the diagnosis of LOS.

## Supplementary Information

**Additional file 1: Table S1. **Characteristics of infants in the sepsis group and control group.

## Data Availability

The data and materials supporting the conclusions of the study are available from the corresponding author on reasonable request.
