# Supplementary material for: The potential value of plasma receptor interacting protein 3 in neonates with culture-positive late-onset sepsis
Source: BMC Infect Dis. 2021 Sep 6;21:919. doi: 10.1186/s12879-021-06636-0 (PMC8422743; doi:10.1186/s12879-021-06636-0)
Supplement: Supplementary file 1 — Additional file 1: Table S1. Characteristics of infants in the sepsis group and control group. [file 12879_2021_6636_MOESM1_ESM.docx]

**Additional file 1 Table 1.** Characteristics of infants in the sepsis group and control

| Characteristics | Sepsis group (*n*=63) | Control group (*n*=79) | *p* value |
| --- | --- | --- | --- |
| **Sex**  Male  Female | 44(69.8%)  19(30.2%) | 47(59.5%)  32(40.5%) | 0.202 |
| **GA (weeks)**  <28  28-<32  32-<37  ≥37  **Median GA (weeks)** | 6(9.5%)  24(38.1%)  19(30.2%)  14(22.2%)  32.1(30.1-36.6) | 7(8.9%)  26(32.9%)  34(43.0%)  12(15.2%)  33.3(30.4-35.3) | 0.425  0.767 |
| **Delivery mode**  Vaginal delivery  C-section | 33(52.4%)  30(47.6%) | 54(68.4%)  25(31.6%) | 0.052 |
| **APGAR score (< 7 )** |  |  |  |
| at 1 min  at 5 min | 4(6.4%)  0(0%) | 8(10.1%)  0(0%) | 0.549  >0.999 |
| **Amniotic fluid-pollution** | 1(1.6%) | 3(3.8%) | 0.629 |
| **Birth weight (g)** | 1550(1250-2800) | 1600(1250-2200) | 0.957 |
| **Pregnancy status**  Multiple pregnancy  Spontaneous pregnancy  GDM  PROM (≥ 18 h) | 12(19.1%)  58(92.1%)  6(9.5%)  11(17.5%) | 19(24.1%)  69(87.3%)  9(11.4%)  9(11.4%) | 0.473  0.363  0.719  0.302 |
| Prenatal fever | 2(3.2%) | 3(3.8%) | 0.796 |
| **Age at blood collection (d)** | 13(10-22) | 13(7-19) | 0.242 |
| **Laboratory results**  WBC (× 10^9^/L)  PLT (× 10^9^/L)  hs-CRP (mg/L)  **Blood culture**  Gram-positive isolates only  Staphylococcus epidermidis  Staphylococcus hominis  Staphylococcus haemolyticus  Staphylococcus capitis  Staphylococcus aureus  Streptococcus agalactiae  Leuconostoc lactis  Arthrobacter wallichii  Gram-negative isolates only  Escherichia coli  Klebsiella pneumonia  Serratia marcescens  Parabacteroides distasonis  Enterobacter aerogenes  Mixed bacterial isolates  Staphylococcus haemolyticus and Staphylococcus epidermidis  Staphylococcus epidermidis and Staphylococcus hominis | 7.6(3.5-10.3)  189(111-289)  7(1-31)  35(55.5%)  17(27.0%)  3(4.8%)  1(1.6%)  10(15.9%)  1(1.6%)  1(1.6%)  1(1.6%)  1(1.4%)  26(41.3%)  10(15.9%)  13(20.6%)  1(1.6%)  1(1.6%)  1(1.6%)  2(3.2%)  1(1.6%)  1(1.6%) | 9.2(6.9-10.7)  301 (216-376)  1(1-2.1)  0(0%)  -  -  -  -  -  -  -  -  0(0%)  -  -  -  -  -  0(0%)  -  - | 0.002  ＜0.0001  ＜0.0001 |

Note: data presented as n (%), mean ± SD or median (IQR)

GA: gestational age; GDM: gestational diabetes mellitus; PROM: premature rupture of membranes; WBC： white blood cells; PLT: platelet; hs-CRP: hypersensitive C-reactive protein.
